# Value of integrated PET-IVIM MRI in predicting Ki-67 expression in newly diagnosed prostate cancer

**DOI:** 10.1038/s41598-025-02467-0

**Published:** 2025-07-01

**Authors:** Liu Xiao, Yuhao Li, Yikai Xing, Lin Li

**Affiliations:** 1 Department of Nuclear Medicine, West China Hospital of Sichuan University, No. 37. Guoxue Alley, Chengdu, 610041 Sichuan People’s Republic of China; 2 West China School of Medicine, West China Hospital, Sichuan University, Chengdu, Sichuan People’s Republic of China

**Keywords:** Prostate cancer, IVIM, SUVmax, Immunohistochemistry, Cancer, Molecular biology, Medical research, Urology

## Abstract

Accurate assessment of Ki-67 expression in patients with prostate cancer (PC) is paramount. Therefore, this study aimed to assess the value of integrated Gallium-68(^68^Ga)-prostate-Specific membrane antigen-11 (PSMA) Positron Emission Tomography/Intravoxel Incoherent Motion Magnetic Resonance Imaging (PET/IVIM MRI) in predicting Ki-67 expression in newly diagnosed PC. A retrospective analysis was conducted on 37 newly diagnosed PC patients who underwent ^68^Ga-PSMA-11 PET/MR for staging. Maximum Standardized Uptake Value( SUVmax) and IVIM parameters of lesions were quantified. Patients were stratified into low-risk (Ki-67 < 5%) and high-risk groups (Ki-67 > 5%). SUVmax and IVIM parameters were compared between the two groups. Of the 37 patients, 29 were categorized as high risk, while 8 were classified as low risk. The high-risk group exhibited significantly higher SUV_max_ (21.4 ± 11.3 vs. 11.2 ± 8.5, *P* = 0.025) and lower Standard apparent diffusion coefficient (ADC) (0.0011 ± 0.00023 vs. 0.0014 ± 0.00039, *P* = 0.005) compared to the low-risk group. Receiver operating characteristic (ROC) analysis determined optimal cut-off values for predicting high-risk patients as 7.64 for SUV_max_ (sensitivity: 96.6%, specificity: 59.1%) and 0.0013 for standard ADC (sensitivity: 89.7%, specificity: 52.3%). Integrated assessment of SUVmax and standard ADC using ^68^Ga-PSMA-11 PET-IVIM MRI may aid in predicting Ki-67 expression, with optimal thresholds of 7.67 for SUVmax and 0.0013 for standard ADC. These findings offer novel insights into evaluating the biological behavior of prostate cancer tumors in patients undergoing PET/MR imaging.

## Introduction

Prostate cancer (PC) stands as the second most prevalent cancer and the foremost cause of cancer-related mortality among men worldwide. The inherent biological heterogeneity of PC manifests in varying clinical behaviors, spanning from manageable “low-risk” disease to formidable “high-risk” lethal cancers^1^. Despite PC’s relatively high survival probability, its propensity for protopathic mortality remains at approximately 10%. Therefore, the imperative to identify and validate biomarkers capable of prognosticating disease outcomes becomes paramount, facilitating clinicians in refining treatment strategies. Tumor differentiation and proliferative activity emerge as pivotal predictors of biological behavior, with the marker Ki-67 serving as a gauge for the proliferation of PC cells, intricately linked with epithelial-mesenchymal transition^2^. Studies have revealed that patients exhibiting low Ki-67 expression tend to experience prolonged progression-free survival (PFS) and overall survival (OS), whereas upregulation of Ki-67 correlates with heightened PC aggressiveness^3,4^. Presently, Ki-67 assessment typically involves preoperative biopsies or postoperative immunohistochemical analyses, underscoring the critical need for accurate non-invasive evaluation of Ki-67 levels.

Intravoxel incoherent motion (IVIM), founded on the dual-exponential model, facilitates the assessment of two distinct motion states of water molecule diffusion and blood flow perfusion via multi-b imaging^5^. Prior investigations have demonstrated the utility of IVIM parameters, such as apparent diffusion coefficient (ADC), in discerning Gleason grade variations^6^. Furthermore, IVIM-related parameters have progressively found application in preoperative assessment of Ki-67 expression across diverse tumor types^7,8^. Positron emission tomography(PET)/Magnetic resonance imaging (MRI) incorporating IVIM sequences represents a novel multiparametric imaging modality capable of concurrently providing insights into metabolism, diffusion, and perfusion. Integrated PET-IVIM MRI has been leveraged to evaluate phenomena such as lymphovascular space invasion,^9^ lymph node metastasis,^10^ as well as the expression of hypoxia-inducible factor-1α (HIF-1α) and vascular endothelial growth factor (VEGF)^11^, while also facilitating tumor response assessment and recurrence prediction^12,13^. Maximum standardized uptake value (SUV_max_) derived from Gallium-68(^68^Ga)-prostate-Specific membrane antigen-11 (PSMA) PET scans can be considered a surrogate marker for Ki-67 expression, particularly in patients displaying positive PSMA expression^14^. As immunohistochemical analysis remains the gold standard for Ki-67 assessment, the development of non-invasive predictive methods using integrated PET-IVIM MRI could provide valuable preoperative information for clinical decision-making. Hence, the present study endeavors to assess the utility of integrated ^68^Ga-PSMA-11 PET-IVIM MRI in predicting Ki-67 expression in newly diagnosed prostate cancer cases.

## Methods

### Study population

A retrospective cohort comprising 37 newly diagnosed prostate cancer patients was assembled for this study. All participants underwent ^68^Ga-PSMA-11 PET/MR imaging for staging purposes. Inclusion criteria encompassed individuals identified as men with histologically confirmed prostate carcinoma and devoid of any prior therapeutic interventions such as chemotherapy or pelvic radiotherapy. Exclusion criteria included the presence of other malignant tumors or systemic diseases, as well as contraindications for PET/MR scanning (e.g., metallic implantation, intrauterine device [IUD], or claustrophobia disorder). The proliferative index (Ki-67 expression) was determined by immunohistochemical analysis.

### PSMA PET-IVIM image

All imaging procedures were conducted utilizing a multimodal PET/MR system (GE Signa PET/MR). Following an intravenous injection of 0.055 mCi/kg ^68^Ga-PSMA-11, participants underwent pelvic PET/MR imaging subsequent to a one-hour delay. PET and MRI scans were performed simultaneously. A standard MR protocol, incorporating T2-weighted, T1-weighted, and diffusion-weighted imaging (DWI), was executed for all subjects. Intravoxel incoherent motion (IVIM) imaging was acquired utilizing 12 b-factors (b value = 0, 10, 30, 50, 80, 100, 150, 200, 400, 600, 800, 1000, 1200 s/mm2) with three diffusion-encoding gradient directions, allowing up to three averages. IVIM data acquisition utilized multi-slice Spin Echo (SE) with axial single-shot echo planar imaging (EPI) employing the following parameters: field of view (FOV) = 320 × 320 mm2; matrix size: 128 × 128; voxel size: 2.5 × 2.5 × 4 mm3 with a 0.2 mm slice gap; repetition time (TR) = 3.500 s, echo time (TE) = minimum ms. Subsequent post-processing of the IVIM data was conducted utilizing the GE AW Volume Share 7 workstation. PET imaging comprised a 10-minute acquisition time per bed position within the pelvis. All PET images underwent reconstruction utilizing an ordered subset expectation maximization algorithm, incorporating parameters such as time-of-flight (TOF) modeling, 2 iterations, 28 subsets, matrix size = 192 × 192, slice thickness = 2.8 mm, field of view (FOV) = 60 cm, and filter cut-off = 5 mm.

### Image analysis

**Fig. 1:**
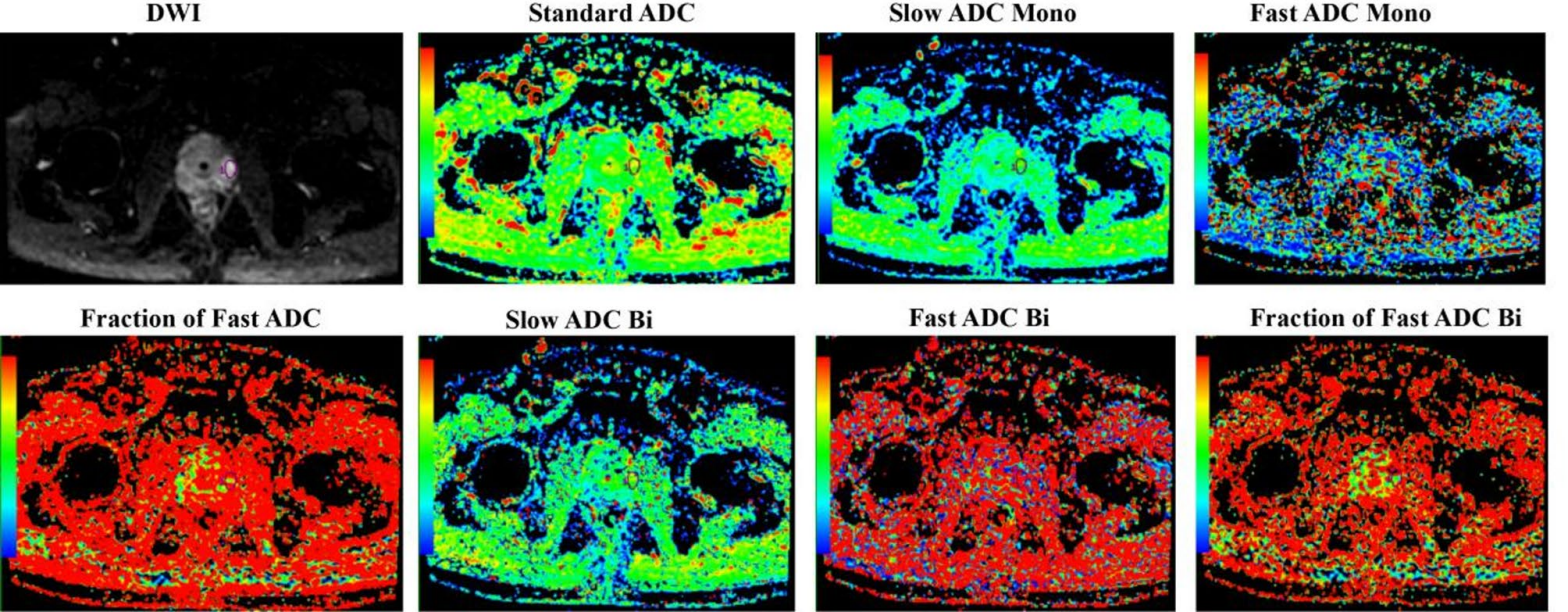
Relevent IVIM parameters.

The maximum standardized uptake value (SUV_max_) of each lesion was quantified for every patient on ^68^Ga-PSMA-11 PET/CT scans. Tumor volumes of interest (VOIs) were manually delineated by two experienced nuclear medicine physicians(LX and YL) on ^68^Ga-PSMA PET/MR images with reference to T2-weighted MRI to ensure anatomical accuracy. SUV_max_ was automatically derived as the highest voxel value within the defined VOI, normalized to injected dose per body weight. IVIM parameters included Standard apparent diffusion coefficient (ADC), Slow ADC Mono, Fast ADC Mono, Fraction of Fast ADC Mono, Slow ADC Bi, Fast ADC Bi, and Fraction of Fast ADC Bi, as delineated in Fig. 1.

### Statistical analyses

Statistical analyses were performed utilizing SPSS 22.0 (IBM SPSS Statistics, USA). Data were reported as mean ± standard deviation (SD) for normally distributed variables; if not normally distributed, they were given as median (range). Differences in SUV_max_ and IVIM parameters were assessed between low and high Ki-67 expression groups. Receiver operating characteristic (ROC) curve analyses were conducted to ascertain the optimal cut-off values of these parameters for predicting high Ki-67 expression. A significance level of *P* < 0.05 was applied for statistical inference.

## Results

A total of 37 PC patients were enrolled in the study. The mean age was 68.3 ± 9.1. Patients were stratified into two groups based on Ki-67 expression levels: low risk (Ki-67 < 5%) and high risk (Ki-67 ≥ 5%), as delineated in previous literature^15^ Of the total cohort, 29 patients fell into the high-risk category, while 8 patients were classified as low risk. Comparative analysis revealed that the high-risk group exhibited elevated SUV_max_ levels (21.4 ± 11.3 vs. 11.2 ± 8.5, *P* = 0.025) and decreased Standard ADC values (0.0011 ± 0.00023 vs. 0.0014 ± 0.00039, *P* = 0.005) compared to the low-risk group. No statistically significant differences were observed in the other IVIM parameters between the high and low-risk groups (Table 1).

**Table 1:** Comparsion of patient characteristic and IVIM parameters between low-risk and high-risk group.

Index	Total patients (*n* = 37)	Low-risk group (*n* = 8)	High-risk group (*n* = 29)	*P*
Age	68.3 ± 9.1	63.7 ± 10.1	69.5 ± 8.6	0.109
tPSA at the time of diagnosis	31.1(15.3–87.8)	9.1(4.5–33.9)	37(19.4–93)	0.076
SUV_max_	19.2 ± 11.5	11.2 ± 8.5	21.4 ± 11.3	0.025
Gleason score	7.8 ± 0.8	7.1 ± 0.4	7.9 ± 0.9	0.026
Standard ADC	0.0012 ± 0.0003	0.0014 ± 0.00039	0.0011 ± 0.00023	0.005
Slow ADC Mono	0.001 ± 0.00103	0.0011 ± 0.00026	0.001 ± 0.0016	0.943
Fast ADC mono	0.0148 ± 0.00804	0.0129 ± 0.00588	0.0153 ± 0.00855	0.45
Fraction of Fast ADC mono	0.2842 ± 0.1145	0.3314 ± 0.11952	0.2712 ± 0.11169	0.192
Slow ADC Bi	0.0009 ± 0.00057	0.0011 ± 0.0003	0.0008 ± 0.00062	0.197
Fast ADC Bi	0.0492 ± 0.02731	0.0484 ± 0.04288	0.0495 ± 0.02233	0.92
Fraction of fast ADC Bi	0.2789 ± 0.09115	0.2806 ± 0.07536	0.2784 ± 0.096	0.952

**Fig. 2:**
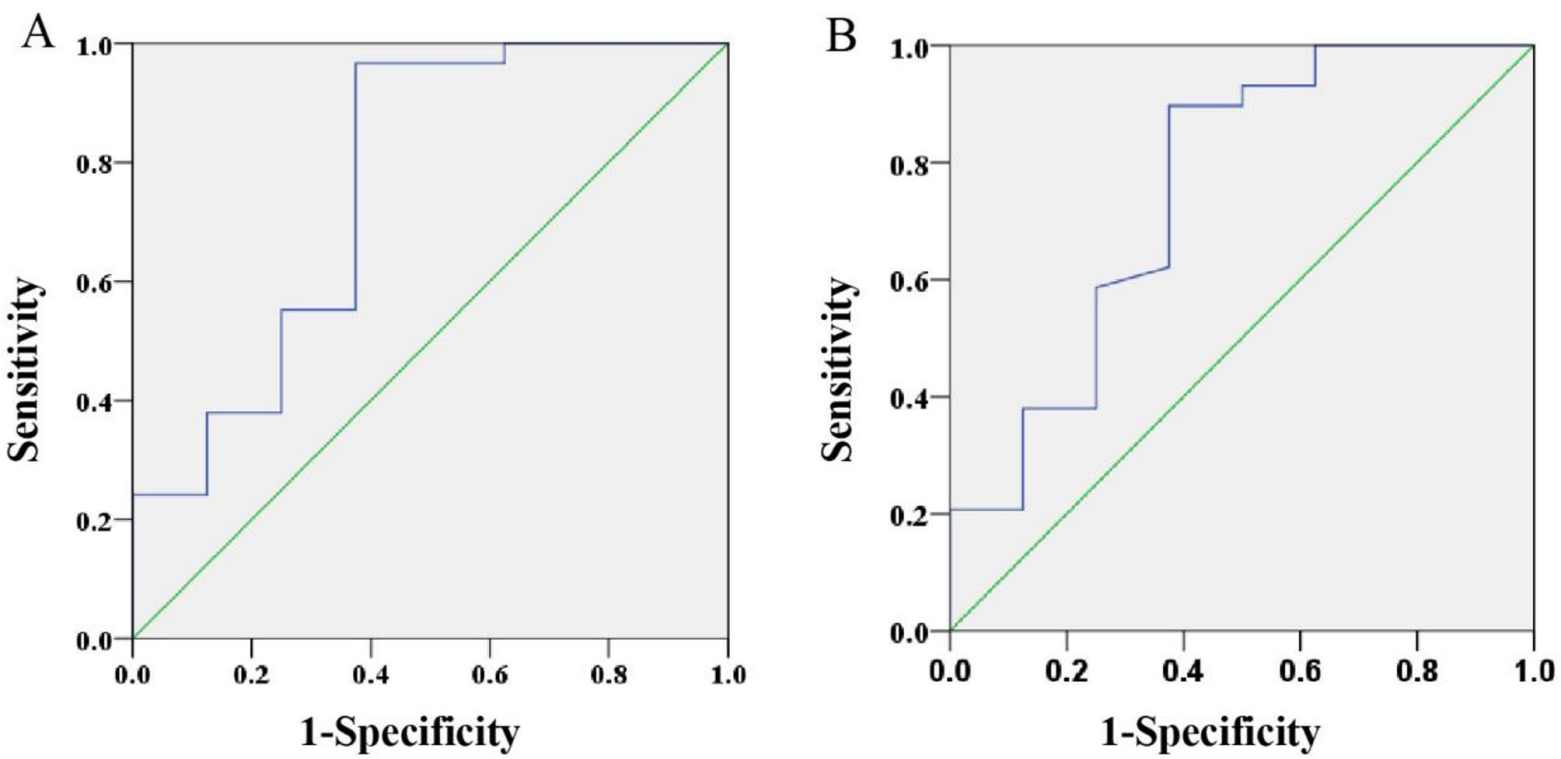
ROC curve showed diagnostic performance of SUV_max_ (A) and standard ADC (B).

**Fig. 3:**
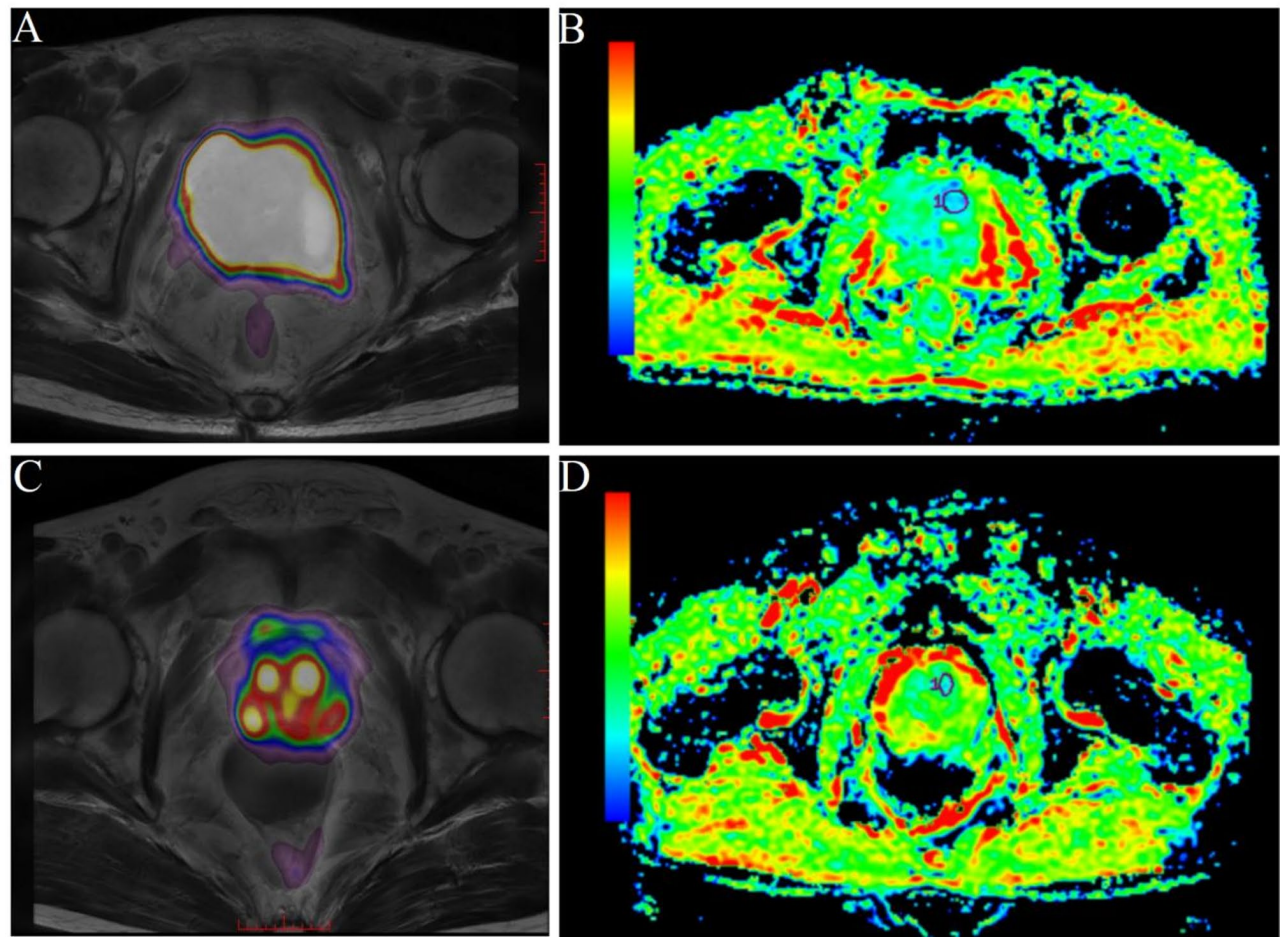
A high-risk prostate cancer patient had positive Ki-67 with 30%. ^68^Ga-PSMA-11 PET/MR showed the lesion had intense focal ^68^Ga-PSMA-11 uptake with an SUV_max_ of 18.34 (A) and corresponding standard ADC value was 0.000731(B). However, a low-risk patient with Ki-67 of 2%, whose SUV_max_ and standard ADC was 7.49 and 0.00135, respectively (C and D).

Next, we conducted ROC curve analysis to determine the optimal cut-off values for predicting high-risk status. As depicted in Fig. 2, the area under the curve (AUC) for SUV_max_ was 0.763 (95% confidence interval: 0.545–0.981; *P* = 0.024), with an optimal cut-off of 7.64 to predict high risk, yielding a sensitivity of 96.6% and specificity of 59.1%. Similarly, the optimal cut-off for standard ADC was determined to be 0.0013, associated with a sensitivity of 89.7% and specificity of 52.3% for identifying the Ki-67 high-expression group. The AUC for standard ADC was 0.752 (95% confidence interval: 0.535–0.969; *P* = 0.031). Notably, three patients exhibited SUVmax values exceeding 7.67 and standard ADC values below 0.0013, all of whom were classified as high risk. Figure 3 illustrates the imaging characteristics of PET/MRI (A and C) and IVIM (B and D) in a high-risk and low-risk prostate cancer patient with Ki-67 expression levels of 30% and 2%, respectively.

## Discussion

In this investigation into the predictive value of integrated PET-IVIM MRI for Ki-67 expression in prostate cancer, our findings highlight the significant potential of standard ADC derived from the IVIM model in conjunction with SUV_max_ from ^68^Ga-PSMA PET for predicting Ki-67 expression in newly diagnosed prostate cancer cases. The identified optimal cut-offs of 0.0013 for standard ADC and 7.67 for SUV_max_ provide valuable insights for clinicians in predicting Ki-67 expression prior to treatment initiation.

Standard ADC, a widely utilized IVIM parameter, reflects the average diffusion characteristics of water molecules within tissues^16^. Our study reveals an inverse correlation between standard ADC and Ki-67 expression in prostate cancer patients. This observation aligns with similar findings reported across various tumor types, including soft tissue sarcoma,^17^ rhabdomyosarcoma,^18^ sinonasal malignancy^19^. Notably, the determined optimal cut-off for standard ADC of 0.0013 exhibited a sensitivity of 89.7% and specificity of 52.3% for predicting Ki-67 expression.Comparable research conducted by Zhang et al. involving 41 soft tissue sarcoma patients to evaluate standard ADC for predicting Ki-67 expression demonstrated a corresponding cut-off of 0.001305, yielding a sensitivity of 77.8% and specificity of 69.6%.^17^ The increase in Ki-67 expression is indicative of heightened tumor cell proliferation, leading to denser cellular arrangements and reduced extracellular space. This phenomenon likely underlies the observed negative correlation between standard ADC and Ki-67 expression^17^.

^68^Ga-PSMA-11 PET/CT has emerged as a cornerstone in the diagnosis, staging, restaging, and prognosis evaluation of prostate cancer. While PSMA is a well-established biomarker for prostate cancer, previous studies have demonstrated the predictive value of SUVmax from ^68^Ga-PSMA-11 PET in assessing Ki-67 expression, particularly in glioma patients, where higher SUVmax values correlate with high-grade tumors^14,20^. This non-specificity highlights the challenge of relying solely on ^68^Ga-PSMA PET for diagnostic specificity. Our study further corroborates these findings, indicating that SUVmax derived from ^68^Ga-PSMA-11 PET/MR, with an optimal cut-off of 7.67, can effectively evaluate Ki-67 expression in prostate cancer patients. Notably, our observation of three patients with SUV_max_ exceeding 7.67 and standard ADC below 0.0013 being classified as high-risk suggests the potential utility of integrating standard ADC and SUV_max_ for more accurate prediction of Ki-67 expression in prostate cancer. These findings underscore the clinical utility of integrated PET-IVIM MRI in facilitating pre-treatment prediction of Ki-67 expression, thereby aiding in treatment planning and decision-making processes for prostate cancer patients. Further validation studies involving larger cohorts are warranted to corroborate and extend these findings, ultimately enhancing our understanding and clinical management of prostate cancer.

The increasing demand for non-invasive imaging biomarkers in oncology underscores the clinical relevance of our findings. Although PSMA expression is not exclusive to prostate cancer, our findings suggest that integrated PET-IVIM MRI can serve as a practical tool for preoperative Ki-67 prediction. This is particularly relevant in clinical settings where immunohistochemical results are delayed or unavailable, potentially aiding in timely treatment decisions. The integration of PET and IVIM MRI not only improves diagnostic accuracy but also provides a potential surrogate for Ki-67 assessment, which could reduce reliance on invasive biopsies in selected cases. Despite these promising findings, our study possesses certain limitations. Firstly, the sample size is relatively modest, warranting further prospective studies to validate the efficacy of integrated standard ADC and SUVmax in predicting Ki-67 expression. Secondly, our analysis solely focused on exploring the relationship between IVIM parameters, SUVmax, and Ki-67 expression, without delving into prognostic implications. Future investigations could delve into whether standard ADC and SUVmax hold predictive value for treatment prognosis in prostate cancer patients.

## Conclusion

Our study underscores the potential of integrated SUVmax and standard ADC derived from ^68^Ga-PSMA-11 PET-IVIM MRI in predicting Ki-67 expression, with identified optimal cut-offs of 7.67 for SUV_max_ and 0.0013 for standard ADC. These findings offer novel insights into evaluating the biological behavior of prostate cancer tumors using ^68^Ga-PSMA-11 PET/MR imaging, albeit further validation and exploration are warranted to fully elucidate their clinical utility.

## Data Availability

The datasets generated during and/or analyzed during the current study are available from the corresponding author on reasonable request.
